# Draft Genome Sequence of Paraburkholderia sp. UYCP14C, a Rhizobium Strain Isolated from Root Nodules of Calliandra parvifolia

**DOI:** 10.1128/MRA.00173-19

**Published:** 2019-04-18

**Authors:** Mauricio Langleib, Martín Beracochea, María Zabaleta, Federico Battistoni, José Sotelo-Silveira, Elena Fabiano, Andrés Iriarte, Raúl Platero

**Affiliations:** a Departmento de Bioquímica y Genómica Microbianas, Instituto de Investigaciones Biológicas Clemente Estable, MEC, Montevideo, Uruguay; b Laboratorio de Biología Computacional, Departamento de Desarrollo Biotecnológico, Instituto de Higiene, Facultad de Medicina, Universidad de la República, Montevideo, Uruguay; c Facultad de Agronomía, CURE, Universidad de la República, Montevideo, Uruguay; d Departamento de Genómica, Instituto de Investigaciones Biológicas Clemente Estable, MEC, Montevideo, Uruguay; Georgia Institute of Technology

## Abstract

Here, we present the draft genome sequence of strain UYCP14C, a rhizobium isolated from Calliandra parvifolia nodules. The assembled genome size was around 9.8 million bp, containing 9,031 predicted protein-coding sequences, including several symbiotic and nitrogen fixation genes. UYCP14C appears to be a novel species of the plant growth-promoting *Paraburkholderia* genus.

## Abstract (precis)

Here, we present the draft genome sequence of strain UYCP14C, a rhizobium isolated from Calliandra parvifolia nodules. The assembled genome size was around 9.8 million bp, containing 9,031 predicted protein-coding sequences, including several symbiotic and nitrogen fixation genes.

## ANNOUNCEMENT

The genus *Paraburkholderia* is composed of Gram-negative, rod-shaped, straight or slightly curved bacteria that contain one or more flagella. These bacteria are metabolically and morphologically similar to the members of their sister clades *Burkholderia*, *Caballeronia*, and *Robbsia* (1, 2). *Paraburkholderia* comprises diverse environmental and plant-beneficial strains, including dinitrogen-fixing legume symbionts (3–9). In particular, the ability of *Paraburkholderia* spp. to nodulate plants of the genus *Calliandra* has been reported (10). The genome sequences of these rhizobia are valuable resources for understanding the mechanisms and evolution of beneficial plant-microbe interactions (11).

Here, we present the draft genome sequence of the bacterial strain UYCP14C, a rhizobium isolated from root nodules of the native legume *Calliandra parvifolia*, naturally occurring in Rio Negro Department, Uruguay.

Root nodule collection and symbiotic bacterial isolation were performed as previously described (12, 13). UYCP14C identification was performed at EzBioCloud (14) using the PCR-amplified 16S rRNA gene sequence. The most similar sequence (99.49%) corresponded to Paraburkholderia monticola strain JC2948^T^. Genomic DNA was isolated from stationary-phase cultures in liquid tryptone-yeast (TY) medium (15) using the ZR fungal/bacterial DNA kit (ZymoResearch). The whole-genome, paired-end library was constructed using the TruSeq PCR-free kit (Illumina), and samples were run on a HiSeq 2500 sequencer (Illumina) at Macrogen (South Korea). A total of 2.3 × 107 paired-end reads 101 bp long were produced, with 99.66% of the reads having a Phred quality score over 30.

Sequence trimming was performed using Sickle 1.33 (16). The UYCP14C genome was assembled with SPAdes v3.9.0 (17), keeping paired reads unmerged before assembly. The kmer length parameter was set to 21, 33, 55, and 77, and SPAdes was executed with the “–careful” flag, which activates the BayesHammer read correction module (18). Genome assembly quality was assessed with QUAST v5.0.1 (17). The total assembly sums 9.83 megabase pairs, distributed in 427 contigs, with an *N*_50_ contig size of 121.2 kb and a maximum length of 381.2 kb. The average coverage reported by the assembler is approximately 60×. Genome G+C content was 63.07%, which is within the range reported for *Paraburkholderia* species (2).

The genome was uploaded to the RAST v2.0 annotation server under classic annotation scheme (18). In total, 9,031 protein-coding genes and 67 RNA-coding genes were annotated. Genes related to nodulation, such as *nodCDHIJ*, and nitrogen-fixation, such as *nifHDK* (among others), were found.

Pairwise average nucleotide identities were calculated using OrthoANI calculator v1.40 (19) by comparing the UYCP14C genome with the 43 genome sequences of organisms of the *Paraburkholderia* genus available in GenBank and analyzed previously (1). In all cases, the average nucleotide identity (ANI) was below the usual threshold of 95% employed as a minimum to designate members of the same species, suggesting that the UYCP14C genome is the first of a new *Paraburkholderia* species. Further studies are in progress to validate this hypothesis.

### Data availability.

Raw reads are available under the BioSample accession number SAMN10846787. The assembled genome sequence was deposited at GenBank under BioProject accession number PRJNA517875. The version described in this paper is the first version under the accession number SELS00000000.
